# Differential Roles for Parietal and Occipital Cortices in Visual Working Memory

**DOI:** 10.1371/journal.pone.0038623

**Published:** 2012-06-05

**Authors:** Daisuke Matsuyoshi, Takashi Ikeda, Nobukatsu Sawamoto, Ryusuke Kakigi, Hidenao Fukuyama, Naoyuki Osaka

**Affiliations:** 1 Department of Psychology, Graduate School of Letters, Kyoto University, Yoshida-honmachi, Sakyo, Kyoto, Japan; 2 Department of Integrative Physiology, National Institute for Physiological Sciences, Okazaki, Aichi, Japan; 3 Department of Psychology, Graduate School of Human Sciences, Osaka University, Yamadaoka, Suita, Osaka, Japan; 4 Human Brain Research Center, Kyoto University Graduate School of Medicine, Shogoin-kawaharacho, Sakyo, Kyoto, Japan; 5 Department of Physiological Sciences, The Graduate University for Advanced Studies, Shonan Village, Hayama, Kanagawa, Japan; University of Regensburg, Germany

## Abstract

Visual working memory (VWM) is known as a highly capacity-limited cognitive system that can hold 3–4 items. Recent studies have demonstrated that activity in the intraparietal sulcus (IPS) and occipital cortices correlates with the number of representations held in VWM. However, differences among those regions are poorly understood, particularly when task-irrelevant items are to be ignored. The present fMRI-based study investigated whether memory load-sensitive regions such as the IPS and occipital cortices respond differently to task-relevant information. Using a change detection task in which participants are required to remember pre-specified targets, here we show that while the IPS exhibited comparable responses to both targets and distractors, the dorsal occipital cortex manifested significantly weaker responses to an array containing distractors than to an array containing only targets, despite that the number of objects presented was the same for the two arrays. These results suggest that parietal and occipital cortices engage differently in distractor processing and that the dorsal occipital, rather than parietal, activity appears to reflect output of stimulus filtering and selection based on behavioral relevance.

## Introduction

The number of representations one can simultaneously hold in visual working memory (VWM) is highly limited; behavioral studies have suggested that it is up to about four items in humans [1], [2]. This limitation has been thought to make the brain prioritize the processing of relevant over irrelevant information [3], [4], [5], [6], [7], [8]. Maintaining a limited number of representations in an active state by sustained attention plays a primary role in VWM, which is considered to be the interface through which attentional control mechanisms filter and select information from cluttered environments [9], [10]. Visual attention and VWM are intimately linked [9], [10], [11], although the exact sameness between the two is questioned [12], [13].

Recently, electrophysiological and neuroimaging studies have demonstrated that this capacity-limited memory system resides in the posterior parietal and occipital cortex [14], [15], [16]. The activity of the IPS has been assumed to reflect the number of representations held in VWM because the activity shows memory load-dependent responses [14], [16], [17], while some parts of occipital cortices have also been known to show similar memory load-dependent responses [14], [15], [16], [18]. It remains, however, to be demonstrated how differently each memory load-dependent area contributes to VWM, especially when task-irrelevant items are to be excluded.

Although working memory is often, because of its severely limited capacity, considered to store only necessary information [3], [6], [7], recent studies suggest that the frontoparietal network encodes not only necessary objects but also unnecessary objects so as to control occipital activity [19], [20]. In fact, Tsushima et al [21] found that representations of distractors in visual areas are not subject to effective inhibitory control when they are subthreshold and not represented in the prefrontal cortex. Furthermore, posterior parietal lesions have been shown to impair the filtering of distractors [22]. These results, together with findings showing that the frontoparietal network biases activity in the earlier visual pathway to enable effective processing of targets and distractors [19], [20], [23], suggest the need for representing distractors in the frontoparietal network to exert inhibitory control over visual areas.

Load-dependent responses in parietal and occipital cortices may thus reflect distinct aspects of attentional control in VWM; i.e., activity in the parietal cortex may reflect the “source” of stimulus filtering and selection, while that in the occipital cortex may reflect the target or output of that control process. Here, we investigated this issue by elucidating which regions are susceptible to task-relevant stimuli, using a change detection task in which participants are required to remember pre-specified targets and ignore distractors [5], [24]. We hypothesized that activity would decrease when items were task-irrelevant in the occipital cortex but not in the IPS, because the source (parietal) region has to handle task-irrelevant stimuli so as to modulate (e.g., suppress) activity in the target (occipital) region.

## Materials and Methods

### Participants

Eighteen university students (eleven females; mean age 23.5 years, range 19 to 31) participated in the experiment [24]. They all had normal or corrected-to-normal visual acuity and normal color vision. All participants received information on fMRI and reported no history of psychiatric or neurological disorders. Each observer gave written informed consent after being apprized of the procedure which had been approved by the Committee of Medical Ethics, Kyoto University Graduate School of Medicine. Data from two participants (two females) with excessive head motion during the scan were excluded from analysis.

### Design and Procedure

**Figure 1:**
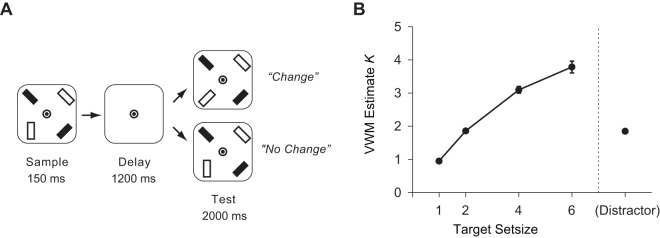
Experimental protocol and behavioral results. ***A.*** An example trial. A trial type with two targets and two distractors (distractor condition) is shown. Participants had to indicate whether a red rectangle changed its orientation, while ignoring blue rectangles as distractors. Stimuli are not drawn to scale. ***B.*** Behavioral results. The behavioral VWM estimate *K* (open circles) and accuracy (filled circles) are shown as a function of trial types. Error bars reflect ±1 SEM.

The experimental design and procedures have been described elsewhere in detail [24] and are summarized here. An example trial is depicted in Fig. 1A. Each 6-s trial started with a sample display containing one, two, four, or six red rectangles, or two red rectangles with two blue rectangles (resulting in five experimental conditions), presented for 150 ms. Each rectangle (approximately 1.8°×0.8°) had one of four orientations (vertical, horizontal, left 45°, right 45°) and was located 3.1° away from the fixation point. Following the sample display, a 1200-ms blank interval, and then a 2000-ms test display were presented. One of the red rectangles changed its orientation for half of the trials and did not change for the other half. Participants were required to indicate whether a red rectangle in a test display changed its orientation or not from a sample display during a test display phase while ignoring blue rectangles as distractors. Each functional run consisted of five experimental conditions and a non-event condition (only the fixation point was presented), with the order of conditions pseudo-randomized within runs. Participants completed four functional runs, each including 12 trials per condition.

We used a standard formula [1] to estimate the number of objects held in VWM for each set size. *K* = (hit rate + correct rejection rate−1)×S, where *K* is the VWM capacity estimate, and S is the display set size of the array.

### MRI acquisition

A Siemens Trio 3T scanner equipped with an 8-channel phased array coil was used to measure blood oxygenation level-dependent (BOLD) cortical activity. Functional images were taken with a gradient echo echo-planar pulse sequence (TR = 2 s, TE = 30 ms; flip angle = 90°). Thirty 3-mm thick axial slices (3 mm×3 mm in-plane, gap 0 mm) parallel to the AC-PC line were acquired for 230 volumes in each run. Following the acquisition of functional images, anatomical 3D T1-weighted images (MPRAGE sequence, 1-mm^3^ voxel, 208 axial slices) and T2-weighted images (fast-spin echo sequence, 1 mm×1 mm in-plane, 30 axial slices) were collected.

### Imaging data analysis

Image data were analyzed with BrainVoyager QX (Brain Innovation, Maastricht, The Netherlands). Preprocessing of functional images consisted of slice acquisition time correction, 3D motion correction, intra-session realignment, spatial smoothing (3D 6-mm Gaussian kernel), linear trend removal, and Talairach space registration [25].

To localize VWM-related ROIs, a multiple regression analysis excluding the distractor condition, with sample display onsets convolved with a canonical hemodynamic function, was performed with regression coefficients for each set size weighted by the VWM estimate *K* of the individual observer for that set size [14]. These contrasts were subjected to a random effects analysis (Bonferroni *P*<0.05, corrected for serial correlation) taking the localizing contrast of each observer as a separate predictor. Then, another multiple regression analysis was conducted with non-weighted regressors defined for each experimental condition. Signal magnitudes of each ROI were derived from beta values of the multiple regression analysis. Instead of performing a separate localizer run, we defined ROIs using conditions embedded within the experimental run. Because the localizing contrast was independent of the distractor condition, main contrasts of interest in the present study (i.e., contrasts between the distractor condition and another) were not biased by the localizing contrast per se.

## Results

### Behavior

A repeated measures analysis of variance (ANOVA) excluding the distractor condition revealed a main effect of display set size (*F*_1.47,22.01_ = 197.79, *P*<0.001; Fig. 1B), and planned comparisons showed that *K* increased as a function of display set size (set size 1, 0.95; set size 2, 1.86; set size 4, 3.10; set size 6, 3.78; *t*_15_ = 33.50, *P*<0.001; *t*_15_ = 12.58, *P*<0.001; *t*_15_ = 5.13, *P*<0.001, respectively, for differences between set size 1 and 2, set size 2 and 4, and set size 4 and 6). Although *K* was not asymptotic, this behavioral function, consistent with a previous study [14], was better described by a quadratic function than by a linear function (*t*_15_ = 3.58, *P*<0.01). Behavioral performance under the distractor condition (1.85) was comparable to set size 2 (*t* values <1, for both *K* and accuracy), despite two distractors being added compared to set size 2. These results are consistent with a behavioral study showing that distractors do not affect VWM accuracy [26].

### Imaging data

**Figure 2:**
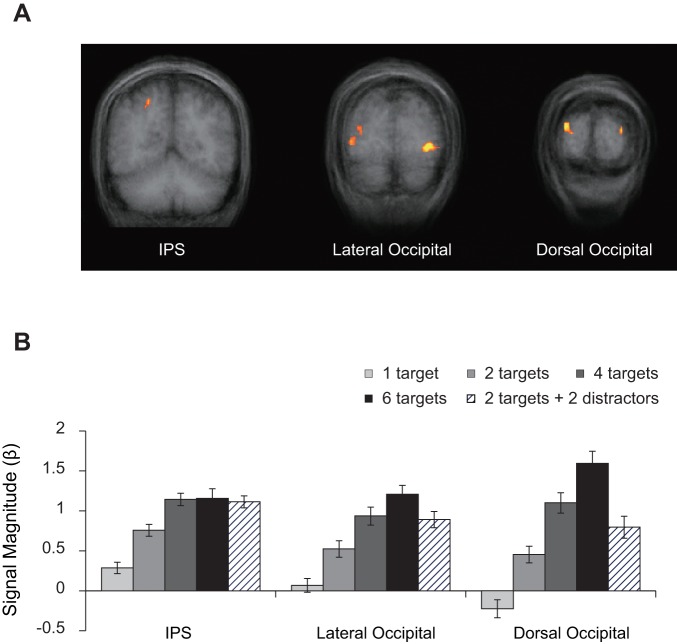
Results of the ROI analysis. ***A.*** Coronal views of the IPS (left, Bonferroni *p*<.05; y = −55), lateral occipital regions (middle, Bonferroni *p*<.05; y = −78), and the dorsal occipital cortex (right, the threshold was ten-fold relaxed from Bonferroni *p*<.05 for the purpose of displaying activation of the left; y = −91). ***B.*** BOLD signal magnitude as a function of trial types in each ROI. Error bars reflect ±1 SEM.

The analysis revealed regions whose activities significantly correlated with the number of objects held in VWM in the right IPS (x = 22, y = −55, z = 46; Fig. 2A left), the bilateral posterior lateral occipital cortex (LO; x = −32, y = −78, z = −4 for left; x = 38, y = −78, z = 1; x = 30, y = −80, z = 10 for right; Fig. 2A middle), and the right dorsal occipital cortex (x = 23, y = −94, z = 11; Fig. 2A right). The left IPS and the left dorsal occipital cortex showed significant activation when the threshold was relaxed twenty-fold and ten-fold, respectively. These regions exhibited qualitatively identical responses with each contralateral counterpart (*F* values <1 for both main effect of hemisphere and interaction between hemisphere and trial type).

BOLD signal magnitude as a function of trial type is shown for each activated area in Fig. 2B. The results for the three activated areas of the LO were pooled for further analysis because there was no significant main effect of area and interaction between area and trial type (*F* values <1). Activities in the LO, and dorsal occipital cortex increased as a function of VWM load (*P* values <001, between set size 1 and 6), while the IPS activity increased up to display set size 4 (*P* values <001 between set size 1 and 4) and thereafter became asymptotic (*t*<1, between set size 4 and 6). The IPS activity was better described by a *K* function than linear function (*t*_15_ = 4.17, *P*<0.001) but this was not true of the LO and dorsal occipital activities (*t* values <1). This suggests the IPS, rather than occipital regions, to be a key neural locus of capacity-limited VWM, but does not necessarily mean the absence of a contribution from the occipital regions to VWM.

Importantly, whereas comparisons between the distractor condition and set size 4 in the IPS and LO areas did not show significance (*t* values <1), the dorsal occipital cortex exhibited a significantly lower response to the distractor condition relative to set size 4 (*t*_15_ = 3.41, *P*<0.01). Differences between the distractor condition and set size 2 were significant in all ROIs (*t*_15_ = 6.39, *P*<0.001 in the LO; *t*_15_ = 5.18, *P*<0.001 in the IPS; *t*_15_ = 2.75, *P*<0.02 in the dorsal occipital cortex).

## Discussion

The present study investigated whether load-dependent responses in parietal and occipital cortices can be differentiated by behavioral relevance. We found that the dorsal occipital cortex showed less of a response to distractors than to targets, while IPS activity did not differ. These results are consistent with the idea that the parietal cortex subserves attentional control and the modulation of occipital activity may reflect output for that control process [19], [22], [27], [28], [29], [30], [31], [32]. The IPS might respond to task-irrelevant items because of the need to manage task-irrelevant information to avoid deleterious effects on behavior. These control processes might cause the modulation of activity in the dorsal visual cortex (see also [5], [20]), although the moderate decline of activity indicates distractor filtering is imperfect.

It is unlikely that the comparable BOLD response between targets and distractors in the IPS is due to the placing of equivalent processing weights on both targets and distractors. Because change detection performance for an array of two targets with two distractors was indistinguishable from that for two targets only, it would seem that participants could effectively prioritize targets and prevent distractors from affecting behavioral performance. Note that this does not necessarily mean perfect distractor filtering: previous behavioral studies, which used a similar change detection task assessed with distractor-change [24] or lure trials [26], have showed that distractors are not filtered perfectly in VWM, while leaving accuracy or memory capacity (*K*) unaffected [24], [26]. This imperfectness of distractor filtering may also be reflected in the dorsal occipital activity. Furthermore, one might argue that items in a test display increased the IPS activity under the distractor condition, because in a test display, more items were present under the distractor condition than two-target condition. However, the IPS showed the same response pattern even when a single item from a sample display was used as a probe instead of all items (see supplemental experiment in [24]). The results thus suggest that the IPS activity is not contaminated by the test display.

The coordinates of the IPS correspond to the superior IPS, which specifically processes featural information, in Xu's superior/inferior distinction of IPS [16]. This may indicate that task-irrelevant featural information is encoded in VWM (but see [33]). Note, however, that it remains unclear whether the IPS activity reflects task-irrelevant representations held in VWM, or if it instead reflects the requirement to focus on targets and/or ignore distractors. The IPS may act as a limited capacity ‘pointer’ system [34] in VWM that can help individuate task-irrelevant objects and help filter them out (see also [22], [35], [36], [37]), or may simply use more attentional resources to concentrate on task-relevant objects or to suppress task-irrelevant objects [38]; both processes are likely to result in IPS activation under the distractor condition. The present results do not distinguish between the two, but nevertheless suggest that, at least to some extent, the IPS processes task-irrelevant information.

The VWM load sensitive activity in the IPS has been considered analogous to the contra-lateral delay activity (CDA) [5], [15], [35], [39], [40]. The CDA is demonstrated to reflect individual differences in allocating VWM capacity [5]: the higher the memory capacity that one has, the more efficiently one can prevent irrelevant items from increasing CDA, i.e., consuming capacity. On the face of it, our results seem to be inconsistent with Vogel et al. [5] in the sense that the memory load sensitive region (i.e., the IPS) showed comparable response to both relevant and irrelevant stimuli. However, as their own scalp topography analysis [39] has shown, CDA is distributed over the parietal and occipital cortex and is computed using relatively lateral/posterior electrodes such as PO3/4. It is therefore possible that their neural evidence for efficient attentional filtering reflected in the CDA originates not only from the IPS but also from the occipital regions as shown by our results^1^. The CDA and the IPS might reflect “somewhat distinct but overlapping” neural mechanisms supporting VWM [41]. In fact, Robitaille et al. [41] has shown that the two neural activities are not necessarily identical. Moreover, in the first place, sulcal activity is difficult to measure in electroencephalogram (EEG) [42], while this is not the case in fMRI. Note that activities in the LO and the dorsal occipital cortex did not reach asymptote at four objects, but this may be due in part to the objects and because the task used in the present study was relatively easy to memorize (see [16], [33]). The LO and the dorsal occipital cortex might have greater processing capacity than the IPS and/or process visual objects in different ways from the IPS.

Although we did not conduct retinotopic mapping and thus can not define the precise retinotopic location, the coordinates of our dorsal occipital activation closely match those of area V3a in a previous study [43]. Recent neuroimaging studies have begun to demonstrate that V3a is involved in figural processing [44], [45], [46]. V3a has a representation of the whole contralateral visual field and a relatively large receptive field, and thus is a likely candidate for early figural integration [44]. In particular, the finding by Scholte et al. [47] that the conscious detection of a segregated figure results in higher V3a responses is suggestive of the susceptibility of V3a to perceptual awareness or attentional manipulations. It therefore seems that the decline in the dorsal occipital activity under the distractor condition reflects attentional modulation of figural representations in the V3a. Although the role of V3a in figural processing is not well understood, the present results may support the notion that V3a is subject to attentional factors.

Finally, the differential responses to distractors we found among VWM load-sensitive regions indicate that each area contributes differently to the processing of task-irrelevant information. Consistent with previous studies [16], [48], we also found VWM load-sensitive activity in the LO. Given that the LO and IPS responded comparably to both targets and distractors, the LO may also contribute to processing of task-irrelevant objects [18], [49], [50], [51]. Note however that the activity of the LO, unlike the IPS whose activity reached asymptote at four objects, tracked the total number of objects in the display. This might reflect general object processing [52], [53], rather than capacity-limited VWM, in the LO. Occipital activations thus would not reflect the VWM capacity-limit itself, but might nevertheless support VWM by processing mid-level aspects of visual objects. Further study will be necessary to understand the particular role and/or the cooperation of intraparietal and occipital regions in the processing of task-irrelevant information (e.g., [54]). The decline of dorsal occipital activity under the distractor condition seems to be consistent with Vogel et al. We could not find, however, a significant correlation between individual capacity estimates (*K*_max_) and encoding task-irrelevant objects into VWM (distractor filtering efficiency: *α*) in the dorsal occipital cortex (*r* = 124). This discrepancy might be because the number of targets and distractors was not necessarily equal between hemifields; e.g., targets were presented in one hemifield and distractors in the other hemifield at times, while targets and distractors were evenly distributed in each hemifield at other times. Alternatively, the CDA and the dorsal occipital activity may reflect different neural mechanisms supporting VWM. Note: Individual capacity estimates were derived from the maximum value of Cowan's *K* across all set sizes of that subject (*K*_max_). Distractor. filtering efficiency was derived from the next formula: *α*  =  (F-D)/(F-T), where α is the filtering efficiency, F is the signal magnitude for the four targets condition, D is the signal magnitude for the two targets with two distractors condition, and T is the signal magnitude for the two targets alone condition [5].
